# P40 The phenotypic and molecular analysis of β-lactam resistance in *Pseudomonas aeruginosa* and *Escherichia coli* samples in Trinidad

**DOI:** 10.1093/jacamr/dlaf118.047

**Published:** 2025-07-14

**Authors:** Reinand Thompson, Patrick Akpaka, Andrew Julien, Aleisha Ali, Chandrashekhar Unakal

**Affiliations:** Department of Paraclinical Sciences, The University of the West Indies, St Augustine, Trinidad and Tobago; Department of Paraclinical Sciences, The University of the West Indies, St Augustine, Trinidad and Tobago; Department of Paraclinical Sciences, The University of the West Indies, St Augustine, Trinidad and Tobago; Department of Paraclinical Sciences, The University of the West Indies, St Augustine, Trinidad and Tobago; Department of Paraclinical Sciences, The University of the West Indies, St Augustine, Trinidad and Tobago

## Abstract

**Background:**

Beta Lactam Resistance is an ever increasing global phenomenon. Tracking both the phenotypic and molecular incidence of β-lactam resistance is of paramount importance.

**Objectives:**

To (i) use AST methods to gain an understanding of the extent of antimicrobial resistance of *Pseudomonas aeruginosa* and *Escherichia coli* in Trinidad and Tobago; and (ii) use PCR to understand the molecular basis of antimicrobial resistance in *P. aeruginosa* and *E. coli* in Trinidad and Tobago.

**Materials and Methods:**

The study collected 132 *Pseudomonas aeruginosa* and 154 *E. coli* and used phenotypic methods to detect antimicrobial susceptibility. The Polymerase Chain reaction (PCR) was used to identify six ESBL genes (TEM SHV, CTX, AmpC, KPC and OXA in the samples.

**Results:**

Of the *E.coli* samples collected, 52 (33.8%) of them were cephalosporin resistant, while among the *P. aeruginosa* samples, 31 (23.5%) of the samples were cephalosporin resistant. 9.2 % of the *E. coli* samples and 23.4 % of the *P. aeruginosa* samples were found to be carbapenem resistant. Of the *Pseudomonas* isolates tested, SHV, CTX, AmpC, OXA and KPC genes were found and within the *E. coli* samples TEM, SHV CTX and OXA genes were found. More than one gene was detected in some isolates.

**Conclusions:**

The results described in this study represent the first and most comprehensive published phenotypic analysis of cephalosporin and carbapenem resistance of both *E. coli* and *P. aeruginosa* in Trinidad and Tobago. The rates of resistance are in line with other studies that have been published internationally. This represents the first time the OXA gene has been described in Trinidad and Tobago and the first time that SHV and CTX type β-lactamase genes have been described in *Pseudomonas* isolates in Trinidad. This study provides opportunity for more research to be conducted on Gram-negative isolates in Trinidad and Tobago and the Caribbean region as many of the tested resistant isolates did not possess any of the genes interrogated, implying that other yet undescribed molecular causes may be responsible. New ESBL and carbapenemase variants have been described in samples showing antimicrobial resistance in Trinidad and Tobago. These findings contribute to the wealth of knowledge of the molecular mechanisms of antibiotic resistance globally but also provide opportunities for further study.

